# Comparative Analysis of Third Molar Segmentation Performance Between Sexes Using Deep Learning Models

**DOI:** 10.3390/diagnostics16070977

**Published:** 2026-03-25

**Authors:** Ayşe Bulut, Melis Büşra Aşkın, Gökalp Çınarer

**Affiliations:** 1 Department of Oral and Maxillofacial Radiology, Physiology, Faculty of Dentistry, Yozgat Bozok University, Yozgat 66100, Türkiye; 2 Department of Orthodontics, Faculty of Dentistry, Yozgat Bozok University, Yozgat 66100, Türkiye; melisaskin@icloud.com; 3 Computer Engineering Department, Yozgat Bozok University, Yozgat 66100, Türkiye; gokalp.cinarer@bozok.edu.tr

**Keywords:** sex estimation, third molar, panoramic radiograph, artificial intelligence, YOLO, segmentation, oral physiology, good health and well-being

## Abstract

**Background/Objectives:** Sex analysis in dental radiographs plays a central role in forensic identification, especially when biological material is compromised or incomplete. While most AI-based studies rely on complete dentition or craniofacial structures, this study investigates whether sex-based information can be extracted solely through segmentation of third molars in panoramic radiographs. **Methods:** A retrospective dataset containing 2818 third molar annotations from 757 panoramic images with balanced class distribution across training, validation, and testing subsets was constructed. Three sample segmentation-based deep learning models—YOLOv12n, YOLO26n, and RT-DETR v2—were evaluated under the same training conditions using detection-focused metrics including sensitivity, recall, and mAP. **Results:** YOLOv12n demonstrated the most balanced performance, achieving the highest mAP@0.50 score of 0.810 and mAP@0.50–0.95 score of 0.574; RT-DETR v2 showed higher sensitivity but lower localization accuracy and significantly longer training time. YOLO26n yielded the highest recall rate but showed an increase in false positives. Class-based analyses indicated sex-specific morphological variability in third molar anatomy, showing consistently higher detection performance in female samples. **Conclusions:** These results demonstrate that isolated third molars encode distinctive sex-related signals and that segmentation-focused frameworks offer an interpretable and clinically relevant alternative to whole-image classification in forensic dentistry. Future studies should incorporate larger multi-population datasets, multi-tooth integration, and explainable AI techniques to further improve robustness and applicability.

## 1. Introduction

Forensic dentistry relies on dental evidence to support human identification, including the estimation of sex and chronological age, in contexts such as mass disasters, homicides, and accidents [1]. Individual identification is a central objective in forensic science, and sex estimation plays a pivotal role by substantially reducing the number of potential matches for unidentified human remains. Sex determination is an essential component of forensic identification, as it reflects differences in growth, maturation, and developmental physiology between males and females. Although DNA analysis is regarded as a highly accurate method for sex determination, its application is often limited by practical constraints, including high cost, time-consuming procedures, dependence on specialized laboratory infrastructure, and reduced reliability when biological samples are degraded, scarce, or mixed. Dentomaxillofacial structures are particularly informative in this context because their formation and maturation follow well-defined physiological processes over time. Moreover, hard tissues such as bones and teeth demonstrate high resistance to environmental damage and frequently preserve their structural integrity under adverse conditions. Previous studies have reported sexual dimorphism in dental and skeletal morphology, suggesting that these structures may serve as accessible and cost-effective indicators for sex estimation. Consequently, the analysis of dental and maxillofacial morphology has become a widely accepted alternative approach in both forensic and clinical contexts [2,3].

Teeth are uniquely suited for sex estimation due to their high resistance to postmortem degradation and their predictable developmental trajectories. Among all permanent teeth, third molars occupy a distinctive physiological position because their development—particularly root formation—continues through adolescence and early adulthood, extending beyond the maturation of other permanent teeth. This prolonged and variable maturation window supports the rationale that third molars can encode subtle sex-related developmental signals, yet such signals may be difficult to capture reliably using conventional, manually performed morphometric approaches. Indeed, traditional morphology-based sex estimation methods are often subjective, poorly repeatable, and time-consuming, requiring multiple labor-intensive processing steps that are susceptible to measurement bias [1,2,4,5].

Panoramic radiographs (orthopantomograms, OPGs) provide a practical imaging modality for radiographic sex estimation based on dental and mandibular structures. Despite inherent limitations, including image distortion, superimposition, and the presence of ghost artifacts, panoramic radiography remains widely used for diagnostic and follow-up purposes. This preference is largely attributable to its cost-effectiveness, widespread availability, rapid acquisition, and relatively low radiation exposure compared with other maxillofacial imaging techniques [6].

Panoramic radiographs allow the visualization of multiple dental characteristics through which sex-related physiological differences in third molar development may be assessed. Radiographically observable features include crown dimensions, root length and morphology, timing of apical closure, and patterns of mineralized tissue deposition within enamel and dentin. Several studies have reported that males tend to exhibit larger tooth dimensions, including greater crown size and increased root length, reflecting differences in overall growth patterns and dental tissue deposition [7,8]. In addition, sex-related variation has been observed in the rate and timing of third molar root development, with differences in the progression of root elongation and apical maturation detectable on panoramic radiographs. Radiographic studies of mandibular third molar root development have shown small but measurable differences in developmental timing between males and females on panoramic images, supporting the idea that sex-related physiological signals are embedded in dental maturation patterns [9,10,11]. Beyond gross morphology, radiographic studies have suggested that differences in mineralization dynamics manifesting as variations in radiographic density and contrast within enamel and dentin may also reflect underlying sex-specific physiological processes during third molar maturation [7,12]. Collectively, these features indicate that panoramic radiographs capture not only static morphological characteristics but also developmental and physiological information related to tooth growth and maturation, providing a basis for identifying sex-related differences in third molars using dental imaging.

Such multi-dimensional and subtle radiographic features are difficult to quantify manually but may be captured through data-driven AI models. The increasing adoption of AI in dental image analysis is largely motivated by the limitations of traditional assessment methods. Conventional radiographic evaluations depend heavily on expert experience and manual measurements, making them time-consuming, observer-dependent, and difficult to standardize, particularly in high-volume clinical or forensic workflows [2,13]. AI-based approaches offer objective, reproducible, and scalable alternatives to traditional image interpretation by minimizing observer dependency and reducing intra- and inter-examiner variability. In contrast to manual assessments, which are influenced by individual expertise and subjective judgment, deep learning models are capable of consistently extracting complex image features directly from radiographic data. Previous studies have demonstrated that AI-driven analysis of panoramic radiographs improves standardization and reproducibility in tasks such as sex estimation and age assessment, while also enabling efficient processing of large image datasets. Moreover, the automated nature of AI-based systems facilitates time-efficient analysis and supports their integration into routine clinical and forensic workflows, particularly in contexts where rapid and reliable decision-making is required [2,3,13,14].

With ongoing technological advancements, artificial intelligence (AI)—particularly deep learning (DL)—is increasingly replacing traditional analytical methods across multiple fields. DL, a subset of machine learning, employs multilayer neural networks to automatically extract complex features from input data, leading to substantial improvements in performance. Convolutional neural networks (CNNs) are especially effective for image analysis [15] and therefore form the backbone of many medical imaging applications. A variety of CNN architectures have been developed to address different analytical needs, including classification-oriented models such as VGG [16], ResNet [17], and DenseNet [18], as well as more computationally efficient networks designed for optimized performance. In addition, object detection and segmentation frameworks, such as Faster R-CNN [19], YOLO [20] and U-Net based architectures [21], extend CNN capabilities beyond classification by enabling simultaneous localization and structural analysis. Collectively, these advances have enabled efficient, scalable, and automated interpretation of medical images while accommodating diverse clinical and computational requirements [6].

Artificial intelligence has also been increasingly integrated into dentistry, supporting a wide range of clinical tasks such as diagnostic imaging, automated detection of oral pathologies, and radiographic interpretation. Beyond diagnostic assistance, contemporary AI systems are being explored for broader clinical applications including prognostic evaluation, treatment planning, and decision-support tools that may assist therapeutic and pharmacological decision making in dental practice [22,23].

Recent advances in artificial intelligence, particularly deep learning, have demonstrated that sex can be estimated directly from panoramic radiographs by capturing complex and non-obvious patterns embedded in dental images, thereby overcoming several limitations of traditional methods. Nevertheless, most existing AI-based studies have relied on global dentition or full dentomaxillofacial information, leaving it unclear whether sex-related physiological information can still be detected when the analysis is restricted exclusively to third molar teeth [1]. Unlike previous studies that rely on whole panoramic images and image-level classification [24], our approach focuses specifically on third molar teeth and integrates object detection-based deep learning models, enabling simultaneous localization and sex classification. This targeted and physiologically oriented strategy provides a more clinically realistic and interpretable framework, particularly in scenarios where complete dentition is unavailable or compromised. While previous studies [25] combined manually measured mandibular and dental dimensions with classical machine learning algorithms on limited sample sizes, the present study employs deep learning-based object detection directly on panoramic radiographs, eliminating the need for manual feature extraction.

This study contributes to the existing literature by demonstrating the effect of sex on segmentation using isolated third molars instead of complete dentition or craniofacial structures, thus expanding the applicability of AI-based methods in dentistry and forensic medicine. Unlike previous approaches, the proposed framework integrates physiologically focused analysis with object detection models, enabling simultaneous localization and classification in panoramic radiographs. A comparative evaluation of YOLO-based and transformer-based architectures provides new insights into how different deep learning paradigms respond to subtle morphological variations in third molars. Overall, this study provides AI-focused analysis of third molars, offering a feasible, clinically relevant, and innovative approach for sex assessment in dental imaging.

Therefore, the aim of this study is to detect and segment third molars from panoramic radiographs using deep learning algorithms and to analyze the results in terms of sex-based performance differences. This study, which limits the evaluation to only third molars, aimed to assess whether analyses could be obtained solely from these teeth, excluding additional teeth or craniofacial structures. It was determined that artificial intelligence models could interpret the segmentation levels of male and female individuals based solely on the radiographic characteristics of third molars, thus evaluating the effect of the sex factor on the detection of third molars in panoramic images.

## 2. Materials and Methods

### 2.1. Study Design and Data Source

Panoramic radiographic data were retrospectively collected from pediatric patients who visited a private dental clinic in Ankara between 2011 and 2024. Eligible individuals were children aged 12–19 years who had previously undergone dental examination and treatment. All radiographic acquisitions were performed using the same orthopantomographic Planmeca ProOne^®^ panoramic system (Planmeca, Helsinki, Finland; Input Line 50/60 Hz, 115–230 V, T16A–T8A) and were obtained by a single operator. For each patient, only a single panoramic radiograph acquired with the same device was included in the analysis.

### 2.2. Image Review and Annotation

**Figure 1:**
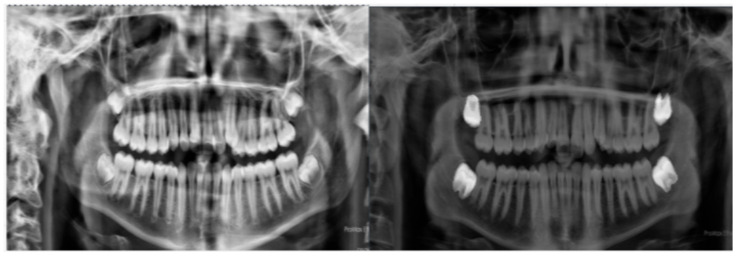
Example dental X-ray images from the ToothData dataset, illustrating male (**left**) and female (**right**) samples.

All radiographs were examined, segmented, and annotated by two experienced dentists from the Departments of Orthodontics and Oral and Maxillofacial Radiology at Yozgat Bozok University Faculty of Dentistry. Annotated tooth masks and bounding regions were converted into structured datasets and used as input for the deep learning pipeline. These annotations served as ground truth for training and validating the model. Figure 1 presents representative images of female and male teeth.

### 2.3. Inclusion and Exclusion Criteria

This retrospective study includes patients who visited a private dental clinic for routine examination or treatment between 2011 and 2024. The study population consisted of individuals aged 12 to 19 years. Eligibility was determined by the presence of at least one diagnostic quality orthopantomogram (OPG). Only radiographs free from imaging artifacts, distortions, or technical deficiencies and providing clear visualization of the third molar regions were included in the analysis.

Patients under the age of 12 were excluded from the study. Additionally, orthopantomographic images exhibiting artifacts, overlaps, or other technical limitations that hindered accurate assessment of relevant anatomical structures were also excluded.

### 2.4. Dataset Split Structure and Class Distribution

Table 1 presents in detail the partitioning of the dataset constructed for segmentation of third molar images into training, validation, and test subsets, as well as the class distribution within each subset.

In total, 2818 third molar labels derived from 757 patients’ panoramic images were included in the analysis, yielding a highly balanced dataset with respect to biological sex (49.75% female and 50.25% male).

The training set comprises 1961 labels derived from 531 patient images, exhibiting a balanced distribution between the female (48.50%) and male (51.50%) classes. This balance reduces the risk of the model developing systematic bias toward either class during training and facilitates effective learning of discriminative morphological features for both sexes. Some images may contain multiple labels or more than one molar tooth. Overall, the fact that class proportions across all dataset partitions remain within the 45–55% range demonstrates that the dataset used for sex-based segmentation on third molar morphology is statistically balanced and methodologically robust. This indicates that model performance is driven primarily variations intrinsic to tooth morphology rather than by class imbalance.

### 2.5. Image Quality Control and Observer Reliability

Image quality criteria were considered throughout the evaluation process. The reliability of assessments was confirmed through systematic procedures, including inter-observer reliability checks, calibration sessions, and consensus meetings between raters. Furthermore, observers were permitted to use image enhancement tools such as contrast and brightness adjustments to optimize visual clarity during review.

### 2.6. Ethics Approval

This study was conducted in accordance with the Declaration of Helsinki (2013 revision) and applicable regulations outlined in the General Health Law. Ethical approval was obtained from the Yozgat Bozok University Ethics Committee (Approval Code: 2025-GOKAEK-255_2025.03.05_318).

### 2.7. Image Preprocessing and Tooth Segmentation

Dental X-ray images present challenges such as low contrast, noise, and the close proximity of anatomical structures. Therefore, image preprocessing and region-of-interest (ROI) localization procedures were applied prior to model training. Each image corresponds to a different patient, enabling the evaluation of the model’s patient-level generalization capability. The task was formulated as instance segmentation, and each tooth region was annotated in YOLO format using the Roboflow [26] platform. All annotation procedures were performed by an expert, and the labels were converted into the YOLO segmentation format. This process enhanced annotation consistency and ensured the integrity of the dataset.

Tooth regions were delineated using rectangular bounding boxes. The ROI is defined as follows:

$$B=\left(x_{m}in,y_{m}in,x_{m}ax,y_{m}ax\right)$$

This approach incurs a lower computational cost compared to the segmentation-based method; however, it also retains background information within the ROI. In this study, the two approaches were evaluated comparatively, and the segmentation-based strategy was observed to achieve superior boundary precision.

### 2.8. Feature Extraction

In this study, feature extraction was performed automatically and in an end-to-end manner using a CNN, rather than relying on manually engineered, classical image-processing-based descriptors. Edge, texture, and shape information of the teeth were learned in a multi-scale manner by the backbone layers of the models. This approach enables more effective representation of the low-contrast and anatomically complex structures characteristic of dental X-ray images.

Within the scope of the comparative analysis, three different deep learning models were employed. These models were selected to evaluate architectural advancements and segmentation performance.

### 2.9. Model Development and Hyperparameters

In this study, three deep learning-based object detection models (YOLOv12, YOLO26, and RT-DETR v2) were employed for sex prediction based on molar teeth. These models were selected to systematically assess the effects of architectural complexity, parameter size, and computational cost on classification performance within the dental imaging context. YOLOv12n and YOLO26n represent lightweight, nano-scale variants of the YOLO family, each comprising approximately 2.5 million parameters. These models were selected due to their efficiency, rapid training times, and suitability for deployment in resource-constrained clinical environments. YOLO26 was selected as the most recent architectural model in this domain, as it is up to date and well suited for performance benchmarking. YOLO26n is a lightweight variant of the YOLO architecture that stands out for its low computational cost and high inference speed. The core designs of these architectures enable rapid convergence while maintaining competitive detection accuracy, making them particularly suitable for real-time or near-real-time dental applications. The YOLOv12n model was included in the comparative analysis due to its single-stage detector structure, optimized backbone architecture, and low memory footprint. YOLOv12, one of the latest technologies in the YOLO series, features a focus on attention-oriented design that pushes the boundaries in real-time object detection. YOLOv12 is compatible with transformers, one of the most researched deep learning approaches today, and features an attention-focused design [27]. Unlike previous CNN-focused YOLO methods, YOLOv12 integrates attention mechanisms while maintaining a high inference rate for real-time object detection [28]. The emergence of RT-DETR [29] opens a new technological avenue for real-time object detection, eliminating dependence on YOLO in this field [30]. In contrast, RT-DETR v2 was included as a transformer-based, high-capacity model with substantially greater representational power, comprising approximately 32.8 million parameters. This model was selected to assess whether increased architectural complexity and global attention mechanisms yield measurable performance gains in sex prediction from molar regions. However, this increased capacity resulted in a markedly longer training time compared with the YOLO-based models. The core characteristics of the models employed in this study are detailed in Table 2. To isolate the effect of the core model architectures from the training configuration, identical or highly similar hyperparameters were applied across all experiments. This approach strengthens the scientific validity of the comparative analyses. The training process was conducted for 50 epochs using an input image resolution of 640 × 640 pixels and a batch size of 16 for YOLO models, 8 for RT-DETR v2 due to GPU memory constraints. Optimization was performed using the multilevel versions of the standard sigmoid (MUSGD) with an initial learning rate set to 0.001667. The YOLO models were trained using the SGD optimizer with momentum of 0.937, weight decay of 0.0005, and learning rate starting at 0.01 with cosine annealing. Pixel intensity normalization was applied using the mean and standard deviation of the training set. RT-DETR v2 was trained using the AdamW optimizer with learning rate 0.0001 and weight decay 0.05. A three-epoch warm-up phase was applied to stabilize the early training dynamics. No data augmentation techniques were applied, in line with the study design focusing on segmentation performance. To ensure reproducibility, a fixed random seed (seed = 0) was used across all experiments. All training procedures were conducted on a CUDA-enabled GPU infrastructure.

No data augmentation techniques were applied during the training process. This decision was intended to preserve the original distribution of the dataset and to ensure that model performance was evaluated solely based on architectural design and learning capacity. In particular, the potential risk of artificial transformations distorting clinical realism in dental X-ray images and consequently biasing model behavior was taken into consideration.

Analysis of the loss functions (box loss, segmentation loss, and classification loss) revealed that all models exhibited stable and monotonically decreasing loss profiles throughout training. No pronounced divergence was observed between the training and validation losses, indicating that the models did not display a tendency toward overfitting. Furthermore, the stabilization of validation performance toward the final epochs of training indicates that the models reached a well-behaved convergence process.

These results demonstrate that the adopted training settings and the dataset effectively supported stable and reliable learning across the models.

### 2.10. Performance Evaluation Metrics

The performance of the models developed in this study was analyzed using widely adopted quantitative evaluation metrics for object detection and segmentation tasks. During the evaluation process, metrics reflecting both classification accuracy and boundary delineation precision were jointly considered.

#### 2.10.1. Precision

The precision metric quantifies the proportion of samples classified as positive by the model that are truly correct. It is a particularly important indicator in clinical applications of this type, where minimizing false positive predictions is critical. Precision is defined as follows:

$$Precision=TP/\left(TP+FP\right)$$

#### 2.10.2. Sensitivity (Recall)

Recall measures the proportion of true positive samples that are correctly identified by the proposed model. In dental imaging, missing tooth regions may have clinically significant consequences; therefore, a high recall value is crucial for model reliability. Recall is defined as follows:

$$Recall=TP/\left(TP+FN\right)$$

#### 2.10.3. F1 Score

The F1 score is defined as the harmonic mean of precision and recall, providing a single-value summary of the balance between these two metrics. It enables a more realistic assessment of model performance, particularly in scenarios where class distributions are not perfectly balanced. The F1 score is computed as follows:

$$F1=2\times \left(Precision\cdot Recall\right)/\left(Precision+Recall\right)$$

#### 2.10.4. Mean Average Precision (mAP)

In this study, model performance was additionally evaluated using the mAP50 and mAP50–95 metrics. mAP50 represents the mean average precision computed at an Intersection over Union (IoU) threshold of 0.50. In contrast, mAP50–95 is a more stringent evaluation metric in which the IoU thresholds are varied from 0.50 to 0.95 in increments of 0.05, thereby providing a more detailed characterization of the model’s boundary delineation accuracy.

These metrics are particularly important in segmentation tasks, as they assess not only the model’s ability to detect objects but also its accuracy in delineating object boundaries.

#### 2.10.5. ROC Curves

Receiver Operating Characteristic (ROC) curves were used to analyze the model’s discriminative capability as a function of varying classification thresholds. ROC curves and the area under the curve (AUC) values were also evaluated in this study; however, Precision–Recall-based metrics were adopted as the primary comparative criteria. This choice was made in consideration of class distribution characteristics and clinical sensitivity requirements in dental X-ray imaging.

## 3. Results

**Figure 2:**
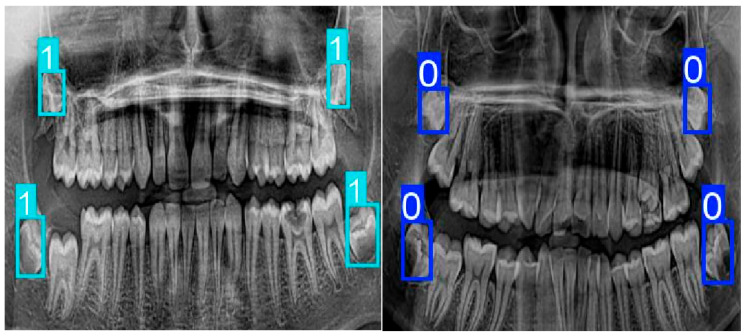
Bounding-Box based visualization of sex prediction results on panoramic dental radiographs (1 = Male, 0 = Female).

When examining the sex prediction results obtained from panoramic dental radiographs in Figure 2, it is observed that the model distinguishes images of male individuals (1) and female individuals (0) using bounding boxes. The detected regions are predominantly concentrated in areas where posterior tooth groups and jaw structures are prominent. This observation indicates that morphological differences between sexes exhibit discriminative characteristics in dental and jaw anatomy. In predictions for male individuals, the bounding boxes were found to be more compact and localized, encompassing the relevant anatomical regions with clearer boundaries. In contrast, predictions for female individuals exhibited bounding boxes that covered broader areas and were positioned to include surrounding anatomical structures. This difference suggests that the model developed a decision mechanism based on distinct spatial features across the sex classes.

Overall, the visual results demonstrate that the deep learning-based model is capable of performing sex prediction from panoramic dental images and successfully learning discriminative features between the classes.

The training time of RT-DETR v2 was approximately 56 times longer than that of the YOLO nano models. This difference can be attributed to its 108 GFLOPs computational demand and its substantially more complex, transformer-based architecture. RT-DETR v2 (L) required substantial computational resources on CUDA-enabled hardware due to its transformer-based architecture.

YOLOv12n and YOLO26n achieved rapid convergence owing to Ultralytics’ latest-generation lightweight architectures.

### Metric Analyses

The results presented in Table 3 summarize the performance of three different object detection models on the sex classification task based on third molars. The models were compared in terms of precision, recall, mAP50, and mAP50–95 metrics.

YOLOv12n demonstrated a balanced performance between precision and recall, achieving 73.8% precision and 80.6% recall. Notably, its attainment of the highest mAP50 (0.810) and mAP50–95 (0.574) values indicates that the model produced more consistent and stable detections across varying IoU thresholds. These findings indicate that YOLOv12n was the strongest model in terms of both localization accuracy and overall generalizability.

RT-DETR v2 achieved the highest precision at 79.0%, making it the model that most effectively controlled false positives. However, its relatively lower recall (77.5%) indicates that some relevant regions were missed. Consistently, the lower mAP50 and mAP50–95 values compared with YOLOv12n suggest that the model’s localization performance was limited, particularly at higher overlap thresholds. This indicates that RT-DETR v2 adopted a more selective but conservative detection strategy. However, YOLO26n’s relatively lower recall (77.5%) indicates that some relevant regions were missed. Its mAP50 (0.778) and mAP50–95 (0.561) values indicate that the model’s overall performance was acceptable, but that the balance between precision and recall was not optimal.

Overall, the YOLOv12n model emerged as the most reliable and balanced approach in this study, owing to its favorable trade-off between precision and recall together with its high mAP values. RT-DETR v2 offers a suitable alternative for scenarios in which controlling false positives is critical, whereas YOLO26n may be preferred in applications where sensitivity is prioritized. These distinct performance profiles indicate that model selection should be optimized according to the intended clinical or forensic use case. Although larger models such as RT-DETR v2 would typically be expected to achieve higher mAP values, the architectural optimizations of YOLOv12n yielded superior results on this specific dataset.

Table 4 presents the confusion matrices of the YOLOv12n, YOLO26n, and RT-DETR v2 models for sex determination based on third molars. Rows correspond to the predicted classes, whereas columns represent the ground-truth labels. In addition to the female and male classes, a background class was included to account for non-dental or diagnostically irrelevant regions. The inclusion of this background class enables a more realistic assessment of model performance in clinical imaging scenarios. RT-DETR v2 achieved the highest correct classification rates for both the female and male classes, whereas the YOLO-based models exhibited higher misclassification between the sex classes and the background category.

The class-wise mAP50 values presented in Table 5 reveal discriminative performance differences of the models for the female and male classes. Across all models, the mAP50 values for the female class were higher than those for the male class. This suggests that the morphological features contributing to sex classification in panoramic dental radiographs of female individuals were learned more consistently by the models.

The **YOLOv12n** model exhibited the highest or most balanced performance across both classes, demonstrating a clear advantage in segmentation for the female class with an mAP50 of 0.837 compared with the other models. Its mAP50 value for the male class (0.783) further indicates that the inter-class performance disparity was limited and that the model possessed strong generalizability.

**RT-DETR v2** achieved highly balanced mAP50 values for the female (0.766) and male (0.778) classes, exhibiting the lowest inter-class variation among the models. This indicates that RT-DETR v2 applied similar decision thresholds across both classes and followed a more consistent, albeit relatively conservative, classification strategy.

For the **YOLO26n** model, the mAP50 value obtained for the female class (0.806) was markedly higher than that for the male class (0.749). This disparity suggests that the model treated anatomical features associated with female individuals as more dominant signals, while exhibiting greater uncertainty for the male class.

Overall, the class-wise results suggest that the female class may exhibit more discriminative characteristics in panoramic dental images, and that this property is directly reflected in model performance. However, the relatively lower mAP50 values for the male class indicate that morphological overlap between sexes and inter-individual variation may complicate the model’s decision-making process. These findings highlight the need for future data and architectural enhancements aimed at improving class balance and strengthening the representation of discriminative anatomical features specific to male individuals.

Examination of the results reveals the occurrence of misclassifications in which a clinically normal male individual was predicted as female in certain regions, while a female individual was predicted as male in others. This indicates that sex prediction from panoramic dental radiographs depends not only on overt morphological differences but also heavily on the spatial and contextual patterns learned by the model.

Although RT-DETR demonstrated superior class discrimination capability between male and female third molars, its lower mAP@0.50 value suggests reduced localization accuracy compared with the YOLO-based models. This discrepancy may stem from reduced sensitivity of the transformer-based architecture to small-scale dental structures and fine anatomical boundaries in panoramic radiographs.

**Figure 3:**
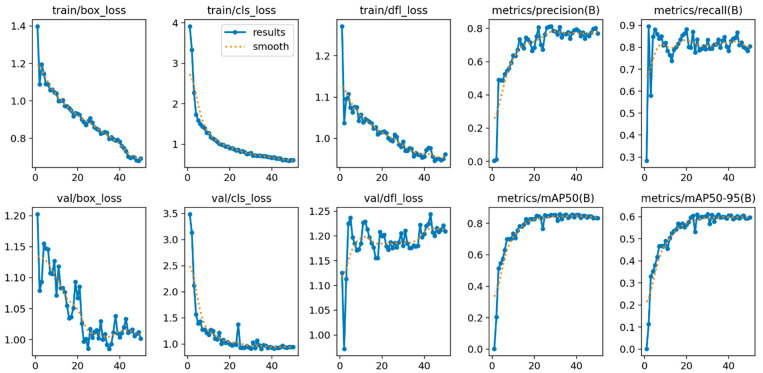
Evaluation of YOLOv12n training and validation curves.

The graphs presented in Figure 3 illustrate the learning behavior and generalization performance of the YOLOv12 model throughout the training process for the sex classification task based on third molar images. The training losses (train/box_loss, train/cls_loss, and train/dfl_loss) exhibited a consistent downward trend across all epochs, indicating that the model learned both object localization and classification tasks in a stable manner.

The gradual decrease in box loss and classification loss during training demonstrates that YOLOv12 progressively improved its ability to accurately localize third molar regions and to discriminate between classes. Similarly, the reduction in distribution focal loss (DFL) indicates that bounding box regression became increasingly precise over time.

Examination of the validation losses shows that the val/box_loss and val/cls_loss curves generally followed a decreasing trend and paralleled the training losses. This indicates that the model did not exhibit pronounced overfitting and that the learned features generalized effectively to the validation data. Nevertheless, the minor fluctuations observed in the val/dfl_loss curve can be attributed to sample diversity and the limited size of the validation set.

From a performance-metric perspective, precision and recall increased rapidly from the initial epochs and reached a stable plateau in later stages of training. This pattern indicates that the model learned fundamental discriminative features at early stages and subsequently refined them in later epochs. In particular, the relatively high recall values suggest a low likelihood of missing third molar regions.

**Figure 4:**
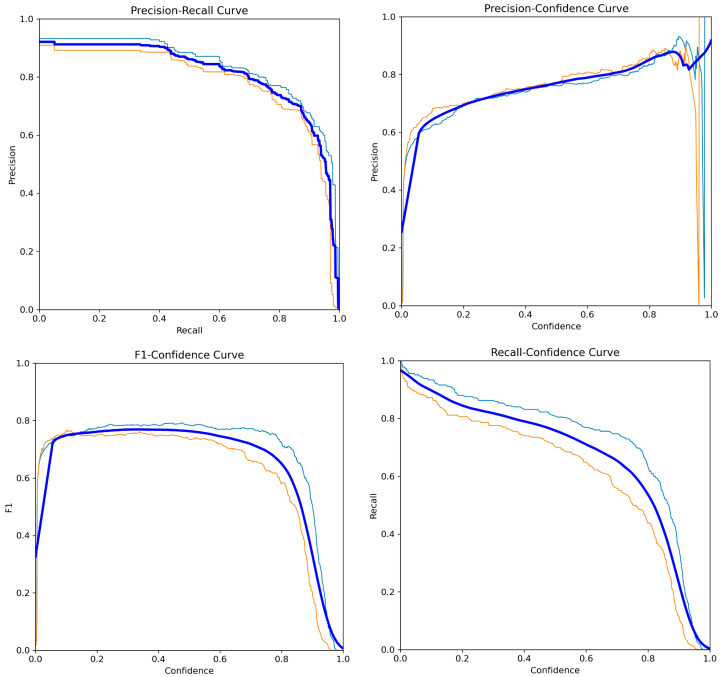
Confidence curves of YOLOv12n on the test dataset.

Finally, the steady and consistent increase observed in the mAP50 and mAP50–95 curves indicates that YOLOv12 exhibited strong and stable detection performance across varying IoU thresholds. The confidence curves of the YOLOv12n model on the test data are presented in Figure 4.

Table 6 summarizes recent AI-based sex estimation studies using dental imaging modalities, highlighting representative image types, analytical pipelines, and deep learning frameworks reported in the literature.

## 4. Discussion

The current study presents a segmentation-based deep learning approach for sex estimation on panoramic radiographs, uniquely restricted to mandibular third molars. Unlike the majority of previous studies summarized in Table 6, which framed sex estimation as a whole-image or multi-region classification task, the present investigation isolates a single dental anatomical landmark to quantify its independent contribution to sexually dimorphic prediction. This methodological refinement offers a valuable lens through which to examine both the capabilities and limitations of AI-driven dental anthropology.

### 4.1. Comparison with Full-Dentition Classification Models

A dominant trend in the literature is the reliance on global dental morphology using full dentition. Multiple studies leveraging convolutional neural networks report high accuracy for sex estimation, routinely surpassing 90%. Ataş [24] achieved 97.25% accuracy using DenseNet121 on full panoramic images, while Hemalatha et al. [32] reported 91.70% performance using a deep CNN pipeline. Similarly, Ciconelle et al. [3] demonstrated that scaling to large, population-diverse datasets (over 200,000 OPGs) allows CNN-based models to capture broad dimorphic cues, achieving up to 95.22% accuracy. These findings collectively underscore that panoramic radiographs encode meaningful sexual dimorphism across a range of dental structures, including mandibular dimensions, crown-root proportions, and bone interfaces measurable at population scale.

More advanced architectures such as EfficientNet variants and hybrid frameworks have further improved outcomes. Hougaz et al. [33] reported accuracies up to 91.43% across several EfficientNet variants, highlighting model sensitivity to feature granularity and network depth. Kim et al. [35] demonstrated that EfficientNetV2 outperformed human observers in sex prediction, and Park et al. [1] attained the highest performance to date (99.20%) with a multi-task model jointly predicting age and sex. Together, these classification-based paradigms demonstrate the upper-bound potential of deep learning when comprehensive dental morphology is available to the network.

### 4.2. Influence of Region Restriction

Against this backdrop, the present study’s segmentation-focused metrics-mAP50 of 0.810 and mAP50–95 of 0.574 appear comparatively lower but must be interpreted within task-specific constraints. Whereas previous models exploited cumulative features dispersed across the mandible and maxilla, the current research interrogates whether third molars alone provide sufficient discriminatory information. Literature on dental and craniofacial morphometry suggests that although third molars exhibit population-level variation, they contribute less consistently to sexual dimorphism relative to broader mandibular skeletal parameters such as ramus width, gonial angle, and mandibular corpus dimensions. This distinction is supported by studies that extracted morphometric features from multiple anatomical indexes rather than isolating a single tooth group including Ortiz et al. [39], who relied on panoramic anatomical reference points, Pertek et al. [37] and Patil et al. [4], who applied mandibular measurements for machine learning-based sex estimation, and Kohinata et al. [36], who demonstrated that whole-jaw profiling yields significantly greater discriminatory power than isolated dental structures. Thus, performance attenuation is anticipated when analysis is anatomically constrained and when models rely solely on third molar morphology rather than mandibular skeletal dimensions.

A small subset of previous AI papers employed limited region-of-interest modeling. For example, Ke et al. [34] introduced a multi-feature fusion network using restricted mandibular regions and reported 94.60% accuracy, demonstrating that reduced feature space can still yield strong discriminative performance when biologically informative structures are emphasized. Ortiz et al. [39] similarly focused on panoramic anatomical measurement points and achieved 89.10% accuracy, validating that non-tooth skeletal cues remain highly predictive. Deep learning studies that automate localization without explicit segmentation, e.g., Bu et al. [2] (ACC 86.79%, AUC 90.64) and Arian et al. [40] (84.49%), further support the notion that reducing usable dental surface area imposes an upper performance ceiling but does not eliminate feasibility, as long as key morphologic landmarks are preserved.

In addition to methodological differences, the present investigation is distinguished from most prior works through its dataset structure and annotation protocol. Unlike studies that relied on hundreds or even thousands of panoramic radiographs across multiple institutions, our dataset consisted of molar images, deliberately curated to ensure standardized positioning and diagnostic quality. Moreover, whereas the vast majority of published models either extracted global image embeddings or annotated a single representative tooth, we manually segmented and labeled all mandibular third molars (teeth 38, 48, 18, and 28) in every radiograph included in the study. This comprehensive annotation strategy maximized the amount of tooth-level information available from each subject despite a comparatively smaller sample size. A further distinction lies in our structured train–validation–test split, which ensured that no subject contributed images to more than one set, thereby mitigating overfitting and enabling unbiased performance estimation. Finally, we implemented and evaluated a single modern object-segmentation architecture, rather than conducting model sweeps across multiple backbones. This choice allowed focused assessment of segmentation-driven inference rather than broad hyperparameter exploration. Together, these design features differentiate the present study from existing literature and provide a controlled framework to specifically interrogate the discriminative power of third molar morphology alone.

### 4.3. Role of Segmentation vs. Classification Paradigms

Few investigations have directly adopted segmentation as the primary predictive interface. Hirunchavarod et al. [43] utilized YOLO-based detectors to automatically localize oral structures and demonstrated mAP@50 values > 0.98, but segmentation served only as a preprocessing step without conversion to biological estimation. Likewise, Kp N. [44] benchmarked multiple YOLO-based pipelines (YOLO-NAS, Roboflow 3.0, YOLOv8), reporting mAP values ranging from 51.55% to 63.60% for sex determination comparable to the performance range observed in the present study further illustrating the intrinsic challenge of learning biological sex exclusively from localized dental tissue. By contrast, most classification-based studies bypass segmentation entirely [14,38] and leverage full radiographic content, explaining their systematically higher performance. These results collectively indicate that segmentation-based pipelines face dual challenges: anatomical boundary extraction and downstream inference. Similarly, in this study, the YOLOv12 model achieved high success thanks to its optimized backbone structure, which enabled more effective learning of fine details and low-contrast areas in dental X-ray images. Furthermore, its single-stage structure, allowing for simultaneous segmentation and object detection, provided a significant advantage. The current findings align with the expectation that compound objectives yield lower numerical outputs than single-label classifiers.

### 4.4. Differential Performance by Sex

Class-wise patterns in the present study noted marginally higher performance in females relative to males. Similar asymmetries were described by Ortiz et al. [39], where male mandibular variability reduced classifier confidence, and Franco et al. [14], who observed greater uncertainty in male predictions across CNN configurations. Conversely, Vila-Blanco et al. [38] demonstrated that splitting training pipelines into male- and female-specific pathways improved predictive consistency and reduced class imbalance effects, suggesting intrinsic asymmetry in morphologic expression. These findings collectively reinforce the need to explore sex-aware or dual-stream architectures for anatomically constrained segmentation tasks.

It should be noted that part of the study population falls within the adolescent developmental period (12–19 years), during which craniofacial growth and dental maturation are still ongoing. Age-related biological maturation may therefore influence certain morphological characteristics observed in dental structures. Nevertheless, third molars are widely used in forensic investigations because their development continues into late adolescence and early adulthood, making them valuable indicators in identification and developmental studies [45,46]. Despite this advantage, the potential influence of developmental stage on the expression of sexual dimorphism should be considered when interpreting AI-based sex estimation results. The balanced distribution of male and female samples across the dataset may partially mitigate this potential bias.

### 4.5. Population, Training Size, and Generalizability

Dataset scale also emerges as a critical determinant of performance across published studies. Ciconelle et al. [3] analyzed over 200,000 images and reported near ceiling-level performance, underscoring the benefit of increased population heterogeneity and morphometric diversity. In contrast, early ANN-based analyses such as Patil et al. [4] (75.00% accuracy), Kohinata et al. [36] (75.50%), and Pertek et al. [37] (83.00%) demonstrate that limited samples and narrow anatomical focus amplify overfitting risk. Thus, the present work’s restricted dataset and reliance on third molars likely heightened sensitivity to interindividual variation, eruption stage, and projection noise.

### 4.6. Forensic Implications and Model Interpretability

Despite these constraints, focusing exclusively on third molars offers meaningful advantages. Third molars erupt later than other teeth and may remain present in young adults when premolars or incisors are missing due to caries or trauma common forensic challenges. Moreover, segmentation-centered models aid interpretability by enabling visibility of the exact structures driving prediction, an increasingly important criterion in medico-legal evidence reporting. Studies such as Hirunchavarod et al. [43] and Arian et al. [40] emphasize explainability modules (e.g., SHAP (SHapley Additive exPlanations) maps, margin mining), highlighting a field-wide shift away from opaque classifiers toward anatomically grounded inference.

### 4.7. Synthesis and Future Directions

In aggregate, the literature summarized in Table 6 demonstrates that full-dentition classifiers consistently outperform region-restricted models. Nonetheless, the present study shows that even highly localized dental structures encode sex-linked variation detectable by modern segmentation networks. Continued advancement will likely depend on hybrid architectures integrating segmentation with classification heads, multi-view fusion Cone Beam Computed Tomography (CBCT) + OPG, longitudinal models linking ontogenetic stages to sex differences, and domain adaptation across ethnic cohorts.

### 4.8. Limitations

The most significant limitation of this study is the lack of external validation using panoramic radiographs obtained from different institutions or imaging devices. Deep learning-based models, particularly in dental panoramic imaging, are known to be sensitive to variations in device-specific contrast, resolution, and noise characteristics. Consequently, the reported performance metrics should be interpreted within the context of a single-center dataset. Therefore, the current findings should be considered a proof-of-concept demonstrating applicability, rather than definitive proof of broad clinical or forensic applicability. The study population represents a single geographic and demographic cohort; therefore, generalizability to diverse ethnic groups, age ranges, and developmental stages is uncertain and requires validation. Furthermore, this study relies solely on third molars extracted from panoramic radiographs, inherently limiting the available morphological information for prediction. Sexually dimorphic features distributed across the entire mandibular and maxilla, such as ramus height, gonial angle, and arch width, were not included and may have limited model accuracy compared to full dentition approaches reported in previous literature. In this study, panoramic imaging is susceptible to projection errors, magnification distortion, and anatomical overlap, which can disproportionately affect segmentation performance, especially when analyzing a single group of teeth. Finally, the dataset size was modest compared to large-scale studies using tens or hundreds of thousands of radiographs, and therefore the model may be susceptible to population imbalance or inter-observer labeling variability. Additionally, this research examined three model architectures, leaving open the possibility that alternative baseline designs or multi-stage frameworks might provide superior performance.

## 5. Conclusions

This study presents evidence supporting the use of a segmentation-based deep learning framework for sex estimation from panoramic radiographs, focusing specifically on mandibular third molars. The YOLO-based model successfully isolated and segmented third molars, achieving accurate detection performance and demonstrating that even localized tooth anatomy contains informative sexual dimorphism detectable through automated image analysis. Consequently, a comparative evaluation of the YOLOv12n, YOLO26n, and RT-DETR v2 models revealed that YOLOv12n demonstrated the most balanced performance for sex classification from third molars, achieving the highest overall mAP@0.50 value (0.810). While RT-DETR v2 showed superior accuracy and reduced background-related misclassifications, its relatively low mAP@0.50 value (0.772) indicates limitations in the accurate localization of third molar regions under varying anatomical and physiological conditions. YOLO26n achieved higher recall values but experienced a decrease in sensitivity, leading to confusion between female and male classes and consequently a decrease in overall detection accuracy. These findings demonstrate that YOLO-based architectures are more sensitive to subtle morphological variations in third molars, while transformer-based models provide more conservative but accurate predictions. Overall, YOLOv12n emerged as the most effective and robust model for practical sex determination applications based on third molars. The results also highlight the feasibility of segmentation-focused workflows that emphasize interpretability and structural localization rather than holistic image classification. The findings contribute to the growing research in the field of dental artificial intelligence and suggest that tooth-specific models can serve as a complementary strategy, particularly in cases where broader anatomical structures are unavailable or compromised. However, further development is needed for such systems to be reliably used in real-world forensic applications.

### Future Work

Future investigations should expand upon this work through several research directions. First, integrating segmentation with downstream classification modules may enable models to leverage both morphological localization and global contextual inference. Hybrid designs such as multi-head architectures or two-stage pipelines could enhance performance without sacrificing interpretability. Second, incorporating larger and more heterogeneous datasets will be essential to evaluate model robustness across population subgroups, age cohorts, and ethnic backgrounds. Cross-population validation and external test sets should be prioritized to address known demographic variability in third molar development. Third, multi-tooth or multi-region segmentation such as combining mandibular molars, canines, or skeletal landmarks should be examined to quantify the incremental predictive value of additional structures. Longitudinal studies assessing developmental changes may further clarify age-dependent morphologic expression of sexual dimorphism. Additionally, explainable AI techniques (e.g., SHAP, Grad-CAM, saliency attribution) may provide valuable insight into the anatomical features driving model predictions, supporting forensic admissibility. Finally, expanding to complementary imaging modalities including CBCT or intraoral scans may improve volumetric understanding and mitigate limitations associated with 3D projection distortion. Collectively, these directions will advance anatomical granularity, predictive reliability, and clinical utility of AI systems in dental sex estimation.

## Figures and Tables

**Table 1:** Sex Classification Dataset Based on Third Molar Tooth Morphology.

Split	Total Patient	Female Labels	Male Labels	Female %	Male %
**Train**	531	951	1010	48.50	51.50
**Valid**	115	226	210	51.83	48.17
**Test**	111	225	196	53.44	46.56
**Total**	757	1402	1416	49.75	50.25

**Table 2:** Core Characteristics of the Proposed Models.

Feature	YOLOv12n	YOLO26n	RT-DETR v2
**Dimension**	Nano	Nano	Large
**Number of Parameters**	~2.5 M	~2.5 M	~32.8 M
**Training Time**	~15 min	~15 min	~14 h
**Architecture Type**	CNN	CNN	CNN + Transformer
**Model Family**	YOLO	YOLO	Transformer-based DETR
**Model Complexity**	Low	Low–Medium	High
**Feature Extraction**	Convolutional	Convolutional	Self-attention
**Computational Cost**	Very low	Low	Very high

**Table 3:** Comparative Analysis of Algorithm Performance.

Model	Precision (P)	Recall^®^	mAP50	mAP50–95
YOLOv12n	0.738	0.806	0.810	0.574
RT-DETR v2	0.790	0.775	0.772	0.544
YOLO26n	0.616	0.831	0.778	0.561
Performance (Mean)	0.714	0.804	0.786	0.559

**Table 4:** Confusion matrices of the evaluated deep learning models for sex determination from third molar teeth.

Model	Predicted/True	Female	Male	Background
YOLOv12n	Female	182	48	42
	Male	38	140	40
	Background	5	8	0
RT-DETR v2	Female	173	55	50
	Male	37	118	44
	Background	15	23	0
YOLO26n	Female	191	49	13
	Male	30	141	14
	Background	4	6	0

**Table 5:** mAP50 Performance of Algorithms by Sex Class.

Class	YOLOv12n (mAP50)	RT-DETR v2 (mAP50)	YOLO26n (mAP50)
**Female**	0.837	0.766	0.806
**Male**	0.783	0.778	0.749

**Table 6:** AI-based sex estimation studies using dental imaging.

References	Imaging	Tooth/Region	Task	Model/Method	Metric(s)	Key Outcome	Sensitivity	Specificity	Precision	F1-Score
Ataş I [24]	OPG	Dentition	Sex estimation	DenseNet121	Accuracy	97.25%	-	96.80	96.80	97.25
Esmaeilyfard et al. [31]	CBCT	Dentition	Sex estimation	NB	Accuracy	92.31%	91.23	92.01	89.83	-
Hemalatha et al. [32]	OPG	Dentition	Sex estimation	Deep CNN	Accuracy	91.70%	100	85.70	-	-
Hougaz et al. [33]	OPG	Dentition	Sex estimation	EfficientNet-B0	Accuracy	-	-	-	-	91.30% ± 0.47
				EfficientNet-B7		-	-	-	-	90.00% ± 0.01
				EfficientNetV2-Small		-	-	-	-	89.10% ± 0.67
				EfficientNetV2-Large		-	-	-	-	91.43% ± 0.67
Ke et al. [34]	OPG	Dentition	Sex estimation	VGG16 + MFF	Accuracy	94.60 ± 0.58%	-	-	-	-
Patil et al. [4]	OPG	Dentition	Sex estimation	ANN	Accuracy	75.00%	-	-	-	-
Kim et al. [35]	OPG	Dentition	Sex estimation	EfficientNetV2	Accuracy	90.20%	-	-	93.10	90.40
Kohinata et al. [36]	OPG	Dentition	Sex estimation	VGG-Net	Accuracy	75.50%	-	-	-	-
Pertek et al. [37]	OPG	Dentition	Sex estimation	ANN	Accuracy	83.00 ± 1.90%	-	-	-	-
Vila-Blanco et al. [38]	OPG	Dentition	Sex estimation	Two-path CNN(female/male)	Accuracy	91.82%/89.09%	-	-	89.65/94.23	-
Franco et al. [14]	OPG	Dentomaxillofacial	Sex estimation	DenseNet121 (TL vs. FS)	Acc/AUC	TL superior 82.18%, AUC 0.91	-	92.20	80.72	80.64
Ortiz et al. [39]	OPG	Dentition	Sex estimation	Neural Network	Accuracy	89.10%	-	-	-	79.00
Arian et al. [40]	OPG	Dentition	Sex estimation	PENVIT	Accuracy	84.49%	-	-	-	-
Bu et al. [2]	OPG	Dentition	Sex estimation	ResNeXt/EfficientNet/ViT	Acc/AUC	Acc 86.79%, AUC 90.64	86.75	-	92.27	89.42
Ciconelle et al. [3]	OPG	Dentomaxillofacial	Sex estimation	CNN (ResNet)	Accuracy	Up to 95.22%	-	-	-	-
Ilic et al. [41]	OPG	Dentition	Sex estimation	Deep CNN	Accuracy	94.30%	-	-	-	-
Park et al. [1]	OPG	Dentition	Sex estimation	ForensicNet (EffNet-B3 + CBAM)	Accuracy	99.20%	99.00	99.30	-	-
Scavassini et al. [42]	OPG	Nasal aperture	Sex estimation	YOLO11m-cls	Accuracy	74.00%	74.00	74.00	74.00	-
Hirunchavarod et al. [43]	OPG	Dentition	Oral part detection	YOLOv5/YOLOv8	mAP@50	0.989/ 0.989	-	-	98.60/98.60	0.987/ 0.985
Kp N [44]	OPG	Specific anatomical landmarks	Sex estimation	YOLO-NAS	mAP	51.55%	-	-	-	-
				Roboflow 3.0	mAP	57.90%	-	-	85.40	-
				YOLOv8	mAP	63.60%	-	-	85.10	-
Present study	OPG	Only 3rd molars	Sex estimation	YOLOv12	mAP@50/mAP@50–95	0.810/0.574	-	-	0.738	-
				YOLO26		0.778/0.561			0.616	
				RT-DETR v2		0.772/0.544			0.790	

Summary of artificial intelligence-based studies investigating sex estimation from dental imaging. Studies are organized according to imaging modality, analyzed dental region, task, and applied model or method. Most previous investigations formulated sex estimation as a classification problem and therefore reported classification-based performance metrics such as accuracy or AUC. In contrast, the present study adopts a segmentation-based approach focusing exclusively on third molar teeth (18, 28, 38, and 48); accordingly, detection-based metrics (mAP@50 and mAP@50–95) are reported only for segmentation-based models.

## Data Availability

The data presented in this study are available on request from the corresponding author. The data are not publicly available due to privacy and ethical reasons.
